# Attitudes and Preferences Regarding Mental Illness and Treatment Among Christian and Religious/Spiritual-Unaffiliated Individuals in the USA

**DOI:** 10.1007/s10943-025-02332-4

**Published:** 2025-05-16

**Authors:** David M. Schultz, Tiffany M. Harrop, Olivia C. Preston, Brian A. Bulla, Taylor R. Rodriguez, Jacob A. Finn, Joye C. Anestis

**Affiliations:** 1 Chicago DBT Institute, Deerfield, IL USA; 2 Missouri Department of Mental Health, Jefferson City, MO USA; 3 Department of Psychiatry, College of Medicine – Jacksonville, University of Florida, Gainesville, FL USA; 4 Rocky Mountain Regional VA Medical Center, VA Eastern Colorado Health Care System, Aurora, CO USA; 5 Department of Psychology, School of Arts & Sciences, Rutgers, The State University of New Jersey, Piscataway, NJ USA; 6 Rehabilitation and Extended Care, Minneapolis VA Health Care System, Minneapolis, MN USA; 7 Department of Psychiatry & Behavioral Sciences, University of Minnesota – Twin Cities, Minneapolis, MN USA; 8 Department of Health Behavior, Society, & Policy, School of Public Health, Rutgers, The State University of New Jersey, Piscataway, NJ USA

**Keywords:** Treatment preferences, Religiosity, Psychotherapy, Christian, Spirituality

## Abstract

Research regarding the role of a religious/spiritual (R/S) identity in psychotherapeutic processes and outcomes is limited. Consideration of client treatment preferences specifically has implications for treatment engagement and retention; however, research on the relationship between preferences and client R/S identification is mixed. The current project included two studies of R/S-unaffiliated (Study 1, *n* = 96; Study 2, *n* = 135) and Christian (Study 1, *n* = 90; Study 2,* n* = 132) participants in the USA. Study 1 investigated differences between these individuals with respect to attitudes toward mental health, preferences for therapist characteristics, and treatment preferences. Study 2 replicated Study 1 in a different community sample and considered how the degree of engagement in R/S services and activities may play a role in therapy-related variables. Results indicated that Christian individuals differed from R/S-unaffiliated individuals in regard to beliefs about the causes of mental illness; preferences for therapists of the same religion, race, and sexual orientation; and preferences for certain therapy tasks (e.g., homework, psychoeducation, learning skills) and types of therapy (e.g., group, family, couples). Results also indicated that preference for same-religion and heterosexual therapists increased as R/S engagement increased. Our findings highlight the importance of R/S in understanding mental health perceptions and treatment preferences and have implications for clinical practice.

## Introduction

The US’ religious or spiritual (R/S) makeup is changing rapidly (Pew Research Center [PRC], 2020). Christianity remains the largest (65%) R/S group in the USA, yet the percentage of Christians in the USA has dropped 12 points in the last decade (PRC, 2020). Meanwhile, the number of Americans who are unaffiliated with a R/S group (i.e., atheist, agnostic, nonreligious, or nonaffiliated with R/S groups) has tripled since the early 1990 s (Cox & Jones, 2017). For many individuals, R/S affiliation is a powerful aspect of personal identity, influencing worldview, coping strategies, and personal choices. The intersection between R/S identity and clients’ mental healthcare decisions seems particularly salient; however, research regarding the integration of clients’ R/S beliefs and/or practices with psychotherapy has largely remained relegated to pastoral counseling. Likewise, researchers have yet to comprehensively investigate the association between clients’ R/S identification and variables known to impact therapeutic outcomes, such as client preferences regarding psychotherapy (e.g., Swift & Callahan, 2009, 2010) and attitudes toward mental health.

Research concerning client preferences for therapy has increasingly focused on demographic factors such as race (e.g., Charles et al., 2020; Rodriguez et al., 2024) and gender (Rodriguez et al., 2024; Schaffner & Dixon, 2003). R/S beliefs constitute an important piece of sociodemographic information to be incorporated into this emerging body of research. Notably, America’s R/S makeup is stratified across generations, with the growth of R/S-unaffiliated individuals most pronounced among young adults (PRC, 2020). Only 49% of Millennials (born 1981–1996) identify as Christian compared to 76% of Baby Boomers (born 1946–1964; PRC, 2020). Likewise, women and individuals of minoritized racial/ethnic groups are a majority within nearly all organized Christian groups in America, while White individuals and men are a majority among R/S-unaffiliated individuals (PRC, 2020). As such, client preferences in therapy may vary across variables such as age, ethnicity, and gender, which, in turn, may be accounted for by general R/S identity. Failure to consider R/S variables may thus result in the misattribution of R/S differences to race or gender differences.

Among Americans who identify as R/S, 65% belong to at least one Christian denomination (PRC, 2020); however, research findings addressing the intersection of Christian self-identification and treatment preferences have been mixed due to the variety of methods, settings, and samples utilized. Mixed findings are particularly common when examining client preferences surrounding discussion of R/S in session. For instance, Saenz and Waldo (2013) found that most clients at a university counseling center had no desire for prayer to be integrated into sessions, even though many respondents reported prayer to be helpful and important in their personal lives. Another study found that R/S-affiliated clients who chose not to discuss R/S matters in therapy reported that such matters were irrelevant to their presenting problems, that they were undecided about their R/S views during therapy, or that they preferred to discuss R/S matters with trusted clergy members rather than mental health professionals (Morrison et al., 2009). In contrast, other samples reported preferences that therapists incorporate client’s R/S beliefs and personal values into therapy (Kelly, 1994). Notably, a study by Quackenbos et al. (1985) found that, although a minority of clients (35%) preferred R/S counseling over secular approaches, a majority (79%) believed that R/S values were an important topic of discussion in therapy. Among clients who have had R/S conversations during therapy, clients were more likely to bring up the topic of R/S rather than the therapist (Morrison et al., 2009). Richards and Bergin (1997) argued that a lack of clinician initiative to address R/S matters may give clients the impression that it is inappropriate for a therapeutic setting, potentially affecting expectations for therapy.

The extent to which clients prefer therapists who match their own R/S affiliation is also relevant. Concern that one’s R/S beliefs (or lack of R/S beliefs) may not be responded to in a supportive and affirming manner by the attending therapist has been cited as a common obstacle to open discussion of R/S (Quackenbos et al., 1985; Rose et al., 2008), possibly explaining why Christian clients have reported a preference for a therapist with similar R/S beliefs (Guinee & Tracee, 1997). However, no evidence indicates that theologically conservative Christians avoid secular therapists because of R/S differences (Guinee & Tracee, 1997). Psychotherapy patients have reported fears that openly R/S therapists may attempt to encourage clients to adopt the therapist’s R/S values, attempt to influence the client’s personal behaviors, or be inflexible in meeting the client’s needs (Lewis & Epperson, 1991); however, such fears can present in the opposite direction, with Christians reporting fears that secular therapy is incompatible with their beliefs and that R/S-unaffiliated therapists may misinterpret, criticize, or ignore personally important beliefs (Keating & Fretz, 1990).

Causal explanations for mental illness and preferences for form of therapy are also important to consider among Christian versus R/S-unaffiliated individuals. Stanford (2007) reported that approximately 30% of Christians with mental illness reported experiencing negative interactions with their church after seeking counsel for mental illness, with approximately 21% of churches discouraging or forbidding the use of psychiatric medication. Likewise, Trice and Bjorck (2006) found that, among high school graduates attending a Pentecostal Bible training school, demonic interference was the fourth-highest ranked cause of depression out of 32 causal items (behind rape, abuse, and loss of spouse). Since most research regarding attitudes toward mental health has not identified or quantified R/S, it is difficult to assess to what degree previous findings regarding causal attitudes toward mental illness generalize to Christian identity.

Overall, little published research has systematically centered around a comparison of Christian and R/S-unaffiliated individuals when it comes to assessing the impact of R/S on client preferences for therapy form, therapy activities, and therapist match and characteristics. Even less research has examined R/S identities and causal explanations for mental health issues. Our objective was to begin addressing this gap by investigating differences between Christian and R/S-unaffiliated individuals with respect to treatment and therapist preferences of potential clients, as well as attitudes on the causes and treatment of mental illness. Although it is important to study all R/S groups, Christian and non-R/S individuals were selected for this study due to their high prevalence in the USA. These findings may inform clinical practice to improve treatment processes and outcomes (e.g., retention, client motivation) for individuals by considering the influence of R/S identification.

## Study 1

Study 1 investigated the extent to which Christian-affiliated and R/S-unaffiliated individuals differ on preferences for sociodemographic match with a hypothetical therapist. We also examined group differences on treatment-relevant features, including attitudes toward mental health or therapist and therapy preferences (e.g., therapy activities). Based on previous work (e.g., Ripley et al., 2001; Schaffner & Dixon, 2003), we hypothesized that Christians would have greater preferences for a therapist of the same R/S beliefs compared to R/S-unaffiliated people. Additionally, as exploratory analyses, we examined group differences regarding attitudes toward mental health (e.g., perceptions of causal explanations, perceptions of stigma), therapist preferences (e.g., matching gender, sexual orientation), and therapy preferences (e.g., therapeutic activities, definitions of treatment success).

### Method

#### Participants and Procedure

The Study 1 sample included 227 adults recruited through Amazon’s Mechanical Turk (mTurk). Participants were paid $4.00 in remuneration. All study procedures were approved by the Institutional Review Board of the University of Southern Mississippi (IRB 15021002); informed consent was obtained from all individual participants included in the study. Seven participants (3.1%) were excluded due to incorrect responses on one or more of four included quality assurance items (e.g., “Select False for this item”) from the survey. The valid sample (*N* = 220) included 90 (40.9%) participants who self-identified their “religion or spiritual practice” as Christianity, 48 (21.8%) as Atheism, 47 (21.4%) as Agnosticism, and one (0.4%) as “non-religious.” We excluded from analyses 31 individuals who reported a non-Christian R/S affiliation (e.g., Islam, Judaism, “other”). Those excluded did not differ from included participants on age, sex, racial/ethnic identification, or sexual orientation (Age, *t*(215) = − 1.17, *p* = 0.242; gender, *χ*^2^(1, *N* = 217) = 0.23, *p* = 0.892; race/ethnicity, *χ*^2^(4, *N* = 217) = 4.30, *p* = 0.367; and sexual orientation, *χ*^2^(3, *N* = 217) = 1.14, *p* = 0.766).

After removing ten additional participants with missing psychotherapy preference data, the final sample used for analyses (*N* = 176) included 92 (52.3%) R/S-unaffiliated participants and 84 (47.7%) Christian participants. Table 1 presents sociodemographic and historical mental health information. R/S-unaffiliated participants were significantly younger and had greater proportions of sexual minorities than Christian participants, while Christian participants had a greater proportion of Black individuals and females (Age, *t*(184) = −3.10, *p* = 0.002; gender, *χ*^2^(2, *N* = 186) = 8.14, *p* = 0.017; race/ethnicity, *χ*^2^(4, *N* = 186) = 14.59, *p* = 0.006; and sexual orientation, *χ*^2^(3, *N* = 186) = 10.96, *p* = 0.012.).

**Table 1:** Demographics and mental health information for study 1 and 2 participants

	Study 1		Study 2	
	Christian (*n* = 90)	Unaffiliated (*n* = 96)	Christian (*n* = 132)	Unaffiliated (*n* = 135)
Age (*M*[*SD*)]	39.99 (1.21)	35.06 (1.04)	38.54 (11.33)	33.84 (10.09)
Male gender (N [%])	28 (31.1%)	76 (50.0%)	71 (52.6%)	64 (48.5%)
*Race/ethnicity (N[%])*				
White	68 (75.6%)	85 (88.5%)	108 (81.8%)	102 (75.6%)
Black	14 (15.6%)	1 (1.0)	13 (9.8%)	8 (5.9%)
Latinx/Hispanic	3 (3.3%)	5 (5.2%)	4 (3.0%)	10 (7.4%)
Asian	5 (5.6%)	4 (4.2%)	4 (3.0%)	8 (5.9%)
Other	0 (0.0%)	0 (0.0%)	4 (3.0%)	3 (2.2%)
Multiracial	–	–	1 (0.8%)	2 (1.5%)
*Sexual orientation (N[%])*				
Heterosexual	88 (97.8%)	83 (86.5%)	125 (94.7%)	121 (89.6%)
Gay	2 (2.2%)	2 (2.1%)	2 (1.5%)	6 (4.4%)
Bisexual	0 (0.0%)	9 (9.4%)	2 (1.5%)	7 (5.2%)
Asexual	0 (0.0%)	2 (2.1%)	3 (2.3%)	0 (0.0%)
Other	0 (0.0%)	0 (0.0%)	0 (0.0%)	1 (0.7%)
*Mental health information (N [%])*				
Received mental health services as child or adolescent	24 (26.7%)	35 (36.5%)	16 (12.1%)	28 (20.7%)
Received mental health services as adult	52 (57.8%)	51 (53.1%)	44 (33.3%)	47 (34.8%)
Hospitalized as child or adolescent	**–**	**–**	2 (1.5%)	3 (2.2%)
Hospitalized as adult	**–**	**–**	9 (6.8%)	5 (3.7%)
Reported receiving mental health diagnosis in lifetime	34 (38.6%)	40 (43.5%)	33 (25%)	23 (17.0%)
*Frequency of engaging in R/S activity (M[SD])*				
Attending a R/S service monthly	**–**	–	2.11 (2.86	0.00 (0.00)
R/S Practice (like praying) weekly	**–**	**–**	3.18 (6.34)	0.67 (0.61)

In Study 1, one participant identified as transgender

#### Measures

*Demographic and mental health treatment history*. Sociodemographic, R/S, and mental health treatment history information was self-reported by participants.

*Mental health attitudes and preferences*. We assessed treatment-relevant attitudes and preferences using an internally developed Life History Questionnaire (LHQ) that has been used in previously published research. All items had Likert-type scale responses based on level of agreement or preference. One item also inquired about the amount of shame one would feel if they were given a mental health diagnosis. This item was used as a proxy for stigma in the analyses.

#### Data Analytic Plan

Hypotheses were tested in a series of one-way Analyses of Covariance (ANCOVAs) or Multivariate Analyses of Covariance (MANCOVAs) examining mean differences between Christian and R/S-unaffiliated participants on mental health attitudes and preferences. Partial eta-squared (partial-$\eta$^2^) was used as a measure of effect size with 0.01 to 0.05 indicating a small effect, 0.06 to 0.13 a medium effect, and 0.14 or above a large effect (Cohen, 1988). Age, gender, racial/ethnic identification, and sexual orientation were entered as covariates.

### Results

#### Mental Health Attitudes

Mean differences on perceived stigma if given a mental health diagnosis ratings did not differ between groups [*F*(1, 169) = 1.99, *p* = 0.160, partial-$\eta$^2^ = 0.01; R/S-unaffiliated, *M* = 2.41*, *SD = 1.47; Christian, *M* = 2.64*, *SD = 1.59]. However, mean differences on causal explanation of mental illness agreement ratings were significantly different [*V* = 0.15, *F*(13, 154) = 0.2.07, *p* = 0.019, partial-$\eta$^2^ = 0.15]. Univariate effects indicated stronger agreement of “moral or religious failings,” “lack of willpower,” “will of God,” and “lifestyle” among Christians (see Table 2). The homogeneity of covariance matrices (HCM) assumption was not met [Box’s *M* = 134.28, *F*(91, 89,673) = 1.36, *p* = 0.013]; thus, the results should be interpreted with caution and are discussed with this caveat in mind.

**Table 2:** Univariate effects of religious affiliation

Dependent variable	Study 1				Study 2			
	*M*(SD)				*M*(SD)			
	Christian	Unaffiliated	F	_*p*_*η*^*2*^	Christian	Unaffiliated	F	_*p*_*η*^*2*^
*Causal explanations for mental health issues*								
Genetics/biologically predisposed	4.33 (1.15)	4.58 (1.04)	.45	.00	4.33 (1.05)	4.53 (1.07)	2.45	.01
Unmet emotional needs	4.28 (1.23)	4.34 (1.13)	.30	.00	4.20 (1.09)	4.43 (1.03)	2.84	.01
Upbringing/childhood experiences	4.78 (1.20)	4.75 (.98)	.04	.00	4.70 (1.06)	4.91 (.95)	.58	.01
Learned bad habits	3.78 (1.26)	3.83 (1.22)	.04	.00	4.04 (1.29)	4.07 (1.19)	.02	.00
Thought processes/ways of thinking	4.63 (1.19)	4.39 (1.15)	2.44	.01	4.18 (1.22)	4.36 (1.10)	1.48	.01
Lack of willpower	3.02 (1.59)	2.57 (1.31)	5.37*	.03	3.03 (1.47)	3.03 (1.47)	.19	.00
Moral/religious failings	2.35 (1.38)	1.70 (1.03)	7.69**	.04	2.70 (1.49)	1.98 (1.39)	18.14***	.06
Will of god	1.89 (1.41)	1.34 (.90)	7.44**	.04	2.35 (1.44)	1.27 (.81)	68.04***	.21
Unlucky experiences	2.67 (1.46)	2.98 (1.44)	.98	.00	2.92 (1.43)	2.81 (1.41)	1.10	.00
Unconscious desires	2.98 (1.42)	3.07 (1.28)	.01	.00	3.23 (1.48)	3.24 (1.32)	.07	.00
Lack of self-knowledge	3.28 (1.50)	3.48 (1.41)	.14	.00	3.48 (1.43)	3.47 (1.30)	.08	.00
Lifestyle	4.11 (1.27)	3.72 (1.31)	5.03*	.03	3.91 (1.35)	4.13 (1.18)	.91	.00
Current relationship conflicts	4.20 (1.35)	4.17 (1.11)	.00	.00	4.23 (1.23)	4.40 (1.17)	.80	.00
Environmental/situational factors	–	–	–	–	4.21 (1.15)	4.27 (1.31)	.04	.00
*Therapist preferences*								
Same gender^a^/male sex^b^	4.14 (1.51)	3.42 (1.65)	6.37*	.04	2.81 (1.31)	2.69 (1.43)	.39	.00
Same race/ethnicity	3.88 (1.75)	3.35 (1.63)	4.93*	.03	3.61 (1.37)	3.18 (1.50)	6.03*	.02
English as primary language	5.54 (.99)	5.15 (1.43)	9.09*	.04	5.00 (1.41)	5.19 (1.44)	3.02	.01
Same sexual orientation^a^/								
Heterosexual^b^	4.01 (1.79)	3.26 (1.57)	8.64**	.05	3.89 (1.59)	3.15 (1.55)	12.51***	.05
Same religious affiliation	3.71 (1.62)	3.49 (1.61)	1.51	.01	3.33 (1.57)	3.33 (1.67)	.00	.00

Study 1 (R/S-unaffiliated, *n* = 89; Christian, *n* = 83). Study 2 (R/S-unaffiliated, *n* = 135; Christian, *n* = 132)

^a^Terminology used in Study 1

^b^Terminology used in Study 2

*p <.05, **p <.01, ***p <.001

#### Therapist Preferences

Mean preference ratings for therapist similarity on sociodemographic features differed across groups [V = 0.08, *F*(5, 166) = 2.79, *p* = 0.019, partial-$\eta$^2^ = 0.08]. Christian-affiliated individuals indicated stronger preference for a therapist of the same gender, same race/ethnicity, English as a primary language, and same sexual orientation, relative to R/S-unaffiliated people (Table 2). Contrary to hypothesis, groups did not differ in preference for a therapist with their same R/S affiliation. Although the HCM assumption was unmet, Box’s M = 36.47, *F*(15, 119,726) = 2.36, *p* = 0.002, a series of Levene’s tests indicated that equal variances can be assumed for most univariate ANOVAs (*p*s ranging 0.002–0.829).

#### Therapy preferences

Mean differences on preference ratings for homework tasks did not differ between groups [*F*(1, 170) = 0.60, *p* = 0.438, partial-$\eta$^2^ = 0.00; R/S-unaffiliated, *M* = 3.36*, *SD = 1.78; Christian, *M* = 3.76*, *SD = 1.76]. Subsequently, there were no significant mean differences on preference ratings for therapy forms [Λ = 0.99, *F*(6, 165) = 0.340, *p* = 0.915, partial-$\eta$^2^ = 0.01], activities [Λ = 0.93, *F*(7, 164) = 0.340, *p* = 0.915, partial-$\eta$^2^ = 0.01], successful outcomes [V = 0.04, *F*(10, 160) = 0.703, *p* = 0.721, partial-$\eta$^2^ = 0.04], or responses to conflict [Λ = 0.95, *F*(6, 164) = 0.1.45, *p* = 0.200, partial-$\eta$^2^ = 0.05]. Note that the HCM assumption was unmet for the successful outcomes [Box’s *M* = 96.59, *F*(55, 95,380) = 1.65, *p* = 0.002], although Levene’s tests indicated equal variances for univariate analyses (all *p*s > 0.05). Nevertheless, due to nonsignificant omnibus *F* tests, between-subject effects of these analyses were not interpreted.

## Study 2

To increase confidence in Study 1’s exploratory findings, we conducted a second study with methodological improvements and relevant considerations. In addition to self-reported R/S affiliation, we also considered degree of religiosity through frequency of engagement of R/S activities on a weekly and monthly basis. Further, we added a measure of help-seeking attitudes as a broader indicator of attitudinal factors related to mental health that may differ across groups. We expected to replicate Study 1 findings and the role of degree of religiosity was exploratory. Consistent with Singer (1997), we hypothesized that help-seeking attitudes would not differ across groups or relate to religiosity indicators.

### Method

#### Participants and Procedure

Study 2 followed the same procedures as Study 1. All study procedures were approved by the Institutional Review Board of the University of Southern Mississippi (IRB CH2-16,102,704); informed consent was obtained from all individual participants included in the study. The full sample (*N* = 304) included 132 (43.4%) participants identifying as Christian, 69 (22.7%) as Agnostic, 60 (19.7%) as Atheist, and six (2.0%) as nonreligious. We excluded 37 (12.2%) participants based on religious affiliations other than Christianity or R/S-unaffiliated. Excluded participants were significantly older and had a greater proportion of females but did not differ based on race/ethnicity or sexual orientation compared with included participants (Sociodemographic variables included age, *t*(43.58) = −2.65, *p* = 0.011; gender, *χ*^2^(1, *N* = 304) = 4.28, *p* = 0.039; race/ethnicity, χ^2^(7, *N* = 304) = 5.23, *p* = 0.632; or sexual orientation, *χ*^2^(4, *N* = 304) = 5.39, *p* = 0.250). Table 1 presents sociodemographic and mental health information. R/S-unaffiliated participants were younger than Christian participants, but did not differ on gender, racial/ethnic identification, or sexual orientation (Age, *t*(260.03) = −3.57, *p* < 0.001; gender, χ^2^(1, *N* = 267) = 0.45, *p* = 0.502; race/ethnicity, χ^2^(7, *N* = 267) = 8.57, *p* = 0.285; or sexual orientation, χ^2^(4, *N* = 26)7 = 8.81, *p* = 0.066).

#### Measures

*Sociodemographic and mental health treatment history*. See Study 1 for description.

*Mental health attitudes and preferences*. See Study 1 for description.

*Degree of religiosity*. Frequency of engagement in R/S activities was assessed via two questions: “How often do you attend a religious service IN A MONTH?” and “How much time do you spend (in hours) engaging in religious practices (like praying) IN A WEEK?” Both items were write-in responses. Christian participants reported attendance at religious services 0–15 times per month, while R/S-unaffiliated participants uniformly reported zero; Christian participants reported engaging in religious practice 0–60 h weekly while R/S-unaffiliated participants reported between 0–7 h (see Table 1).

*Attitudes Towards Seeking Professional Psychological Help – Short Form (ATSPPH; *Fischer & Farina, 1995*)*. The ATSPPH-SF is a 10-item questionnaire assessing beliefs about mental health and seeking mental health treatment. Respondents used a four-point rating scale, ranging from 0 (“Disagree”) to 3 (“Agree”). Higher scores indicate more positive attitudes toward seeking professional help for mental health issues. Internal consistency reliability estimates were strong (α = 0.83).

#### Data analytic plan

Hypotheses were tested using a series of zero-order correlations, ANCOVAs, or MANCOVAs. Unless noted below, all statistical assumptions were met. Zero-order correlations were interpreted based on commonly accepted benchmarks of 0.1 as small, 0.3 as medium, and 0.5 as large (Cohen, 1988). Age was considered as a covariate in all ANCOVA and MANCOVA analyses. To test hypotheses regarding religiosity, analyses were re-conducted with degree of religiosity variables as a covariate. Weekly activities (skew = 8.50, kurt = 94.17), but not monthly services (skew = 3.02, kurt = 10.77), were square root transformed to achieve a more acceptable distribution of scores (skew = 2.24, kurt = 8.40).

### Results

#### Willingness to seek psychological help and attitudes toward mental illness

Zero-order correlations examined associations between degree of religiosity and willingness to seek psychological help. Neither frequency of attending R/S services (*r* = −0.09, *p* = 0.126) or number of hours engaging in R/S activities (*r* = −0.04, *p* = 0.486) were significantly related to ATSPPH-SF scores. ATSPPH-SF mean levels did not differ between groups [*F*(1, 264) = 1.05, *p* = 0.307, partial-$\eta$ ^2^ = 0.01; R/S-unaffiliated, *M* = 19.29*, SD* = 6.08; Christian, *M* = 18.79*, SD* = 5.76]; the effect of affiliation did not change when degree of religiosity was included as a covariate in the model [*F*(1, 261) = 0.13, *p* = 0.718, partial-$\eta$ ^2^ = 0.00]. Anticipated embarrassment/shame if given a mental health diagnosis did not differ between groups [*F*(1, 264) = 2.64, p = 0.105, partial-η^2^ = 0.01; R/S-unaffiliated, *M* = 2.73, *SD* = 1.48; Christian, *M* = 3.03, *SD* = 1.61], including when degree of religiosity was included as a covariate [*F*(1, 261) = 0.49, *p* = 0.484, partial-$\eta$ ^2^ = 0.00]. However, agreement ratings for causal explanations for mental illness significantly differed [V = 0.24, *F*(14, 251) = 5.65, *p* < 0.001, partial-$\eta$ ^2^ = 0.24]. Univariate effects indicated greater endorsement of “moral or religious failings” and “will of God” among Christians (see Table 2). Note that the HCM assumption was not met [Box’s M = 180.12, *F*(105, 218,553) = 1.62, *p* < 0.001]; thus, results should be interpreted with caution. When degree of religiosity was included as a covariate, the omnibus test of affiliation group variable remained significant [V = 0.12, *F*(14, 248) = 2.35, *p* = 0.004, partial-$\eta$ ^2^ = 0.12], although the effect was slightly reduced.

#### Therapist preferences

Zero-order correlations indicated that degree of religiosity indicators relates to preference for a therapist who is heterosexual (services: *r* = 0.24, *p* < 0.001; activity: *r* = 0.26, *p* < 0.001) and has the same R/S affiliation (services: *r* = 0.14, *p* = 0.021; activity: *r* = 0.14, *p* = 0.019). Neither indicator was significantly related to preferences for other therapist sociodemographic features. Preferences for therapist sociodemographic features significantly differed across affiliation groups [V = 0.110, *F*(5, 260) = 2.57, *p* < 0.001, partial-$\eta$ ^2^ = 0.10], indicating greater preference for a therapist of the same race/ethnicity and heterosexuality among Christians (see Table 2). When degree of religiosity was included as a covariate in the MANCOVA, the omnibus effect of the affiliation group variable remained significant [V = 0.09, *F*(5, 257) = 5.19, *p* < 0.001, partial-$\eta$ ^2^ = 0.09]. Although the HCM assumption was not met, Box’s M = 47.67, *F*(15, 283,438) = 3.11, *p* < 0.001, a series of Levene’s tests indicated that equal variances can be assumed (*p*s > 0.05). Because groups did not significantly differ on preference for same R/S affiliation, a priori hypothesized moderation effects were not tested.

#### Therapy preferences

Preference for therapy formats significantly differed across affiliation groups [Λ = 0.86, *F*(6, 259) = 6.82, *p* < 0.001, partial-$\eta$ ^2^ = 0.14]. Univariate effects indicated greater preference for individual therapy among R/S-unaffiliated people and greater preference for group, family, couples, new or experimental therapies, and psychiatric medication among Christians (see Table 3). The omnibus effect of affiliation group remained significant after including the religiosity variables (V = 0.10, *F*(6, 256) = 4.68, *p* < 0.001, partial-$\eta$ ^2^ = 0.10). A difference in preference for homework tasks was observed [F(1, 264) = 4.520, p = 0.034, partial-$\eta$ ^2^ = 0.01; R/S-unaffiliated, *M* = 3.21*, SD* = 1.73; Christian, *M* = 3.57*, SD* = 1.70], with higher levels of homework preference among Christians. However, after covarying for degree of religiosity, the effect was not significant, *F*(1, 261) = 2.05, *p* < 0.154, partial-$\eta$ ^2^ = 0.01). Neither monthly nor weekly religious practices were significant covariates (both *p*s > 0.10).

**Table 3:** Study 2 univariate effects for religious affiliation

Dependent variable	*F*	_*p*_*η*^*2*^	M (SD)	
			Christian	Unaffiliated
*Therapy form preferences*				
Individual therapy	5.31*	.02	5.34 (1.03)	5.56 (1.03)
Group therapy	24.37***	.09	2.48 (1.50)	1.73 (1.22)
Family therapy	27.82***	.10	3.15 (1.66)	2.14 (1.54)
Couples therapy	25.03***	.09	3.45 (1.68)	2.51 (1.64)
Psychiatric medication	4.75*	.03	2.85 (1.78)	2.51 (1.65)
New or experimental therapy	4.71*	.02	2.72 (1.46)	2.42 (1.40)
*Therapy activity preferences*				
Venting about problems	2.64	.01	4.26 (1.49)	4.06 (1.53)
Getting advice from therapist	.97	.00	4.80 (1.45)	4.67 (1.41)
Learning about mental health	10.68**	.04	4.08 (1.69)	3.56 (1.62)
Learning new skills/strategies	7.99**	.03	4.87 (1.50)	4.41 (1.55)
Problem-solving	2.93	.01	4.87 (1.54)	4.56 (1.51)
Client listening—therapist talking	3.72	.01	4.63 (1.44)	4.37 (1.46)
Client talking—therapist listening	12.03**	.04	4.39 (1.45)	3.84 (1.42)

R/S-unaffiliated (*n* = 135), Christian (*n* = 132)

^*^p <.05, **p <.01, ***p <.001

Preferences for therapy activities differed (V = 0.08, *F*(7, 258) = 0.340, *p* = 0.003, partial-$\eta$ ^2^ = 0.08) across affiliation groups. Univariate effects indicated greater preference for “learning about mental health,” “learning new skills/strategies,” and “client-talking-therapist listening” among Christians (see Table 3). The HCM assumption was not met (Box’s M = 69.89, *F*[28, 244,445] = 2.43, *p* < 0.001), but Levene’s tests indicated that univariate analyses met the assumption of equal variances (*p*s > 0.05). After covarying for religiosity, the omnibus effect of affiliation group was not significant (V = 0.5, *F*(7, 255) = 1.76, *p* < 0.001, partial-$\eta$ ^2^ = 0.5) and the effect was reduced. Neither degree of religiosity variables was significant covariates, both *p*s > 0.10.

Finally, definitions of therapy success (V = 0.04, *F*[10, 254] = 1.29, *p* = 0.289, partial-$\eta$ ^2^ = 0.05) and perceived likelihood of responses to discomfort or disagreement with a therapist’s activities (Λ = 0.97, *F*(6, 259) = 1.56, *p* = 0.159, partial-$\eta$ ^2^ = 0.04) did not significantly differ across groups (V = 0.03, *F*(10, 251) = 0.66, *p* = 0.763, partial-$\eta$ ^2^ = 0.03). Based on the nonsignificant omnibus *F* tests, between-subjects effects were not interpreted. For the latter MANCOVA, the HCM assumption was not met, Box’s M = 86.56, *F*(55, 224,651) = 1.51, *p* = 0.009; thus, results should be interpreted with caution. After covarying for degree of religiosity, the omnibus effect of affiliation group on the Conflict variables was significant and small-sized, (Λ = 0.95, *F*[6, 256] = 2.33, *p* = 0.033, partial-$\eta$ ^2^ = 0.05), but neither of the degree of religiosity variables was a significant covariate, *p*s > 0.05. Univariate effects indicated significantly greater preference among R/S-unaffiliated compared to Christians for (1) asking the therapist to explain the reasoning for an activity with which the participant is uncomfortable or disagrees (Christian: M[SD] = 4.84 [1.07], R/S-unaffiliated: M[SD] = 5.02 [1.08]), (2) refusing to do the activity refusing activity (Christian: M[SD] = 3.10 [1.24], R/S-unaffiliated: M[SD] = 3.40 [1.31]), and (3) stopping coming to therapy (Christian: M[SD] = 3.42 [1.50], R/S-unaffiliated: M[SD] = 3.60 [1.35]).

## Discussion

The current studies examined the relationship between R/S affiliation and therapeutic preferences in two samples collected online via mTurk. Study 1 investigated differences between Christian and R/S-unaffiliated individuals on mental health attitudes and preferences. Study 2 replicated findings observed in Study 1 with a larger sample, a measure of help-seeking attitudes, and consideration of degree of religiosity (i.e., monthly religious service attendance, weekly religious activities). The principal aim of these studies was to extend prior research on R/S affiliation and treatment-relevant variables by exploring differences between self-identified Christians and those identifying as R/S-unaffiliated in association with attitudes toward mental health, preferences for therapist characteristics, and treatment preferences. An additional goal of our research was to examine associations between degree of religiosity (i.e., service attendance, hours spent in R/s activities) and therapy-related variables of interest.

Across studies, a clear pattern emerged regarding R/S affiliation and attitudes about mental health. Christian individuals were more likely to report stronger agreement with “moral or religious failings” and “will of God” as causes of mental illness, suggesting a belief that mental disorders may be somewhat attributable to characterological flaws or R/S factors. Interestingly, no differences were observed between groups on shame or embarrassment due to a mental health diagnosis or on attitudes toward seeking psychological help. These findings indicate that Christianity may open an individual to unique beliefs about the etiology of psychological problems (i.e., need to believe in God to view God as a cause of mental illness); however, Christian affiliation and religiosity were not associated with mental health stigma or resistance to seeking psychological help when compared to R/S-unaffiliated people. As such, interventions aimed at stigma reduction or treatment-seeking behavior should target both Christian and R/S-unaffiliated individuals. Assessing for R/S affiliation and practices prior to beginning a therapeutic intervention may provide useful information regarding perceived causes of mental illness, which may be incorporated into case conceptualization and psychoeducational discussions (see Rosmarin, 2018, for an example on how to do this within the context of cognitive-behavioral therapy).

We hypothesized that Christians would evince a greater preference for a therapist of the same R/S affiliation compared with R/S-unaffiliated individuals, but unexpectedly, there were no differences in preferences for a therapist with the same R/S affiliation. However, degree of religiosity was positively associated with stronger preference for a therapist who has the same R/S affiliation in Christian-identifying participants. This indicates that generally Christian and R/S-unaffiliated individuals both equally prefer to work with a therapist with akin R/S beliefs. For Christians, this preference increases as their degree of religiosity increases.

R/S affiliation was associated with a stronger preference for a therapist of the same race/ethnicity and sexual orientation (denoted as heterosexual in Study 2). The sexual orientation result is consistent with extant findings that general religiousness was associated with less accepting attitudes toward gay/lesbian individuals and lower tolerance of those perceived to behave incongruously with typical R/S teachings (Rowatt et al., 2009). Our race/ethnicity finding is consistent with research on preferences for a match between client and therapist on race/ethnicity (Cabral & Smith, 2011; López et al., 1991; Shin et al., 2005). In general, these results may reflect a belief by Christian-affiliated individuals that a match between client and therapist regarding sexual orientation and race may result in fewer misunderstandings within the therapeutic relationship. Preferences such as these may present as a barrier to help seeking, depending on the salience of the preference for a potential client. These preferences may have differential impact depending on the presenting problem, level of disclosure from the therapist, and the point at which clients and therapists discuss preferences or a mismatch (e.g., earlier or later in treatment). Further research is needed to clarify these potential clinical implications.

Differences emerged between the two samples regarding preferences for therapy forms and activities. Specifically, in Study 2, Christians reported a stronger preference for homework tasks, learning about mental health (i.e., psychoeducation), learning new skills/strategies, client-talking-therapist listening, and therapist talking-client listening. Notably, when accounting for degree of religiosity there is no difference in these preferences between Christian and R/S-unaffiliated individuals. This suggests preference for activities regarding homework, psychoeducation, and client-therapist interactions may be associated with religiosity factors rather than strictly Christian affiliation. Further, examination of differences in perceived likelihood of responses to conflict within the therapeutic relationship revealed no differences between Christian and R/S-unaffiliated respondents; however, when accounting for degree of religiosity, a significant, small-sized effect emerged. Findings revealed greater preference among R/S-unaffiliated individuals regarding the following responses to discomfort/disagreement: requesting the therapist explain the reasoning for an activity, refusing to engage in the activity, and terminating therapy. Rosmarin et al. (2009) found that more general R/S factors (i.e., religious denomination) were overall unrelated to symptoms of affective disorders (e.g., depression, worry), whereas more specific religiosity variables (e.g., importance of religion, frequency of engaging in prayer) were predictive of lower levels of psychological distress. Our results suggest that degree of religiosity may also serve as an important, and ultimately more salient, factor than general R/S affiliation when considering treatment preferences. As such, future research examining links between R/S and the therapeutic process may wish to investigate the impact of degree of religiosity. Additionally, our findings highlight important considerations for mental health providers aiming to incorporate factors associated with R/S identity into therapeutic interventions; namely, the degree to which a person identifies as religious and the activities they engage in related to their R/S affiliation may be as/more important to consider within the therapeutic context than the client’s general R/S identity.

Christian individuals also reported a stronger preference for group, family, couples, and experimental treatment along with pharmacotherapy, whereas R/S-unaffiliated individuals more strongly preferred individual therapy. One explanation for these findings may relate to differences in mental health treatment history across the two samples. In Study 1, over half of both Christian and R/S-unaffiliated individuals had received prior mental health treatment, but in Study 2, only one-third of Christian and R/S-unaffiliated respondents endorsed previous services. Our findings may suggest that both Christian and R/S-unaffiliated individuals with therapy experience have fewer mental health treatment preference differences compared to those without therapy experience. For individuals with prior psychotherapy experience, their R/S affiliation may have less impact on their therapy preferences.

### Study Limitations

The current findings must be considered in light of limitations. The study used a convenience sampling method and was limited to individuals with computer use capabilities. Further, the participants identified as Christian or Atheist/Agnostic, and we excluded individuals who endorsed a non-Christian R/S affiliation (e.g., Islam, Judaism), and those who did not specifically describe being R/S-unaffiliated. We combined all individuals identifying as Christian into a single group rather than separate denominational groups. This broad group classification represents a notable limitation in the interpretation of this data. While approximately 65 percent of individuals in the USA identify as Christian (PRC, 2020), attitudes toward mental illness and therapy preferences are likely to vary across Christian denominations (Dimmick et al., 2020), limiting the degree to which the present study can draw broad conclusions about Christians as a collective whole. Similar limitations may also be seen in other diversity research using broad group classifications (e.g., LGBTQ +, Asian American) which contain substantial diversity within them. Future studies are therefore recommended to take a more fine-grained approach to examining these affiliations by focusing on differentiation between R/S affiliation utilizing larger samples to increase representation. We were unable to examine associations between Christian and R/S-unaffiliated individuals and different types of stigma (e.g., experienced stigma, internalized stigma) that may be useful when targeting mental health-related stigma. However, our findings provide a foundation to build upon in subsequent research exploring R/S affiliation and mental health stigma and help-seeking behavior.

Our multivariate analyses violated the homogeneity of covariance matrices (HCM) assumption, indicating that (1) variances in each dependent variable differ between groups, (2) the correlation between any two dependent variables differs between groups, or (3) both. However, Box’s test may be less reliable with relatively equal sample sizes, and Pillai’s statistic V is robust in adjusting for the violation (Field, 2013). Tabachnick and Fidell (2012) note lower confidence in interpreting HCM violations among smaller samples, such as in the present study. From a statistical standpoint, these considerations increase confidence in our findings, and the HCM violations suggest several relevant avenues for further research. Considering the averages of standard deviations of groups, the violated assumptions open the door to several possibilities. Christian-affiliated may have slightly greater variability in causal explanations for mental illness, while R/S-unaffiliated individuals may have slightly greater variability in therapist preferences. Alternatively, or in conjunction with these possibilities, the correlation between dependent variables may be stronger within groups. For example, Christian-affiliated individuals may have a stronger relationship between preferences toward similar gender and sexual orientation/heterosexuality relative to R/S-unaffiliated individuals. Age may play a role in differences in group variance, with significantly older individuals in the Christian-affiliated group relative to the R/S group in both samples. Taken together, the violated HCM assumptions suggest some heterogeneity in each group relative to different considerations for psychotherapy preferences. This may be addressed in future research through examining differences between denominations in Christianity, exploring differences in attitudes toward mental illness specifically in individuals that do not affiliate with religion or spirituality, testing the impact of age on therapy preferences in conjunction with religious or spiritual affiliation, or through methodological approaches, such as removing or aggregating dependent variables with high correlation.

## Conclusions

The present study contrasted mental health attitudes and treatment preferences for Christian and R/S-unaffiliated individuals. Our findings suggest that Christian individuals reliably varied from those identifying as R/S-unaffiliated in regard to beliefs about the causes of mental illness and preferences for a therapist of the same race and same sexual orientation as themselves. Further, greater degree of religiosity was associated with stronger preference for a therapist of the same R/S beliefs. Assessment of client R/S affiliation provides a meaningful starting point for shared decision-making and treatment planning conversations. Integration of these findings in treatment planning could contribute to greater therapeutic alliance and increased treatment engagement, which may improve clinical outcomes.
